# Assessing Agreement between miRNA Microarray Platforms

**DOI:** 10.3390/microarrays3040302

**Published:** 2014-12-12

**Authors:** Niccolò P. Bassani, Federico Ambrogi, Elia M. Biganzoli

**Affiliations:** Department of Clinical Sciences and Community Health, University of Milan, Via Vanzetti 5 20133, Milano (MI), Italy

**Keywords:** miRNA microarray, reliability, agreement, Bland–Altman, measurement error

## Abstract

Over the last few years, miRNA microarray platforms have provided great insights into the biological mechanisms underlying the onset and development of several diseases. However, only a few studies have evaluated the concordance between different microarray platforms using methods that took into account measurement error in the data. In this work, we propose the use of a modified version of the Bland–Altman plot to assess agreement between microarray platforms. To this aim, two samples, one renal tumor cell line and a pool of 20 different human normal tissues, were profiled using three different miRNA platforms (Affymetrix, Agilent, Illumina) on triplicate arrays. Intra-platform reliability was assessed by calculating pair-wise concordance correlation coefficients (CCC) between technical replicates and overall concordance correlation coefficient (OCCC) with bootstrap percentile confidence intervals, which revealed moderate-to-good repeatability of all platforms for both samples. Modified Bland–Altman analysis revealed good patterns of concordance for Agilent and Illumina, whereas Affymetrix showed poor-to-moderate agreement for both samples considered. The proposed method is useful to assess agreement between array platforms by modifying the original Bland–Altman plot to let it account for measurement error and bias correction and can be used to assess patterns of concordance between other kinds of arrays other than miRNA microarrays.

## 1. Introduction

MiRNAs are small non-coding RNA molecules that have been shown to play a critical role in tumorigenesis [1,2,3,4] and in several other pathologies [5,6,7,8]. In order to measure miRNA intensity levels, several methods, such as RT-qPCR, high-throughput sequencing and microarrays, have been developed and have enabled researchers to profile a large number of miRNAs simultaneously across different experimental conditions [9]. MiRNA microarrays, in particular, since their first appearance in 2004 [10], have known a considerable expansion in life sciences and are now routinely used in biomolecular research.

In the last few years, miRNA microarrays have been compared with next generation sequencing technologies to study their performances [11,12]. The main interest lies in the potentialities and advantages offered by these new platforms in terms, for instance, of new miRNAs discovery [13]. However, since results from these comparison studies appear to be contrasting, miRNA microarrays still remain an effective and useful technology, whose characteristics need to be properly assessed, both in terms of within-platform reliability and of between-platforms agreement.

To date, only a few studies have attempted to evaluate within-platform [14] and between-platform [15,16] reliability in miRNA microarrays. Results were reported mainly as correlation coefficients (both Pearson and Spearman) for evaluating both intra-platform and inter-platform performance, calculated on a subset of miRNA that depended on detection calls concordant between platforms (*i.e.*, miRNA that were called “detected/present” on all platforms considered for the analysis). Additionally, Sato and colleagues assessed between-platform comparability also in terms of miRNAs that were commonly differentially expressed between samples for all platforms [15], whereas Yauk *et al.* [16] evaluated within-platform reproducibility via Lin’s concordance correlation coefficient [17].

In this work, a different approach based on the Bland–Altman method to assess between-platform agreement is proposed. In particular, the proposed method is applied to assess agreement between three different miRNA microarray platforms (Affymetrix, Agilent, Illumina). Additionally, advice against the use of Pearson/Spearman correlation coefficients is provided, and use of concordance correlation coefficients (pairwise and overall) is suggested as a better measure to evaluate within-platform reliability.

## 2. Experimental Section

### 2.1. Samples

The two samples involved in the study are a renal tumor cell line named A498 (ATCC, Manassas, VA, USA) [18] and a pool of twenty different human normal tissues (namely, hREF), obtained from the First Choice^©^ Human Total RNA Survey Panel, (Ambion Inc, Austin, TX, USA). RNA material was analyzed in different laboratories, as follows: Affymetrix processing took place at the Biomedical Technologies Institute of the University of Milan (Segrate, Italy); Illumina and Agilent processing were performed at the Department of Experimental Oncology of the National Cancer Institute (Milan, Italy). For both samples, three technical replicates for each platform were performed, leading to a total of 18 arrays, six for each different microarray platform.

### 2.2. Sample Preparation and Hybridization

The cell lines were cultured at cell confluence according to the corresponding ATCC datasheets. Total RNA samples were extracted using the miRNeasy kit (Qiagen Spa, Hilden, Germany) and quantified by ND-1000 spectrophotometer (NanoDrop Technologies, Wilmington, DE, USA). Using an Agilent 2100 BioAnalyzer (Agilent Technologies, Santa Clara, CA, USA), RNA integrity was assessed on the basis of the RIN (RNA integrity number) factor, and the presence of low molecular weight RNA (5S) was verified. Labeling of total RNA samples was performed using the FlashTagTM Biotin RNA labeling Kit (Genisphere Inc., Hatfield, PA, USA) starting from 1 *μ*g of total RNA. Briefly, the tailing reaction is followed by ligation of the biotinylated signal molecule to the target RNA sample. The labeling reaction is based on Genisphere proprietary 3DNA dendrimer signal amplification technology. Prior to array hybridization, total RNA labeling was verified using the enzyme-linked oligosorbent assay (ELOSA). After that, biotin-labeled samples were hybridized onto the arrays at $48{\;}^{{}^{\circ}}C$, then washed and stained using the Affymetrix Fluidics Station 450. Arrays were scanned with the GeneChip^©^ Scanner 3000 7G to acquire fluorescent images of each array and analyzed by use of GeneChip Operating Software (GCOS, version 1.2).

### 2.3. Data Pre-Processing

#### 2.3.1. Affymetrix GeneChip^©^ miRNA Array

Intensity .CEL files were obtained from the scan images and imported to the Affymetrix^©^ miRNA QC Tool software (Version 1.0.33.0) to quantify the signal value. Quality control (QC) was assessed by plotting the average intensity of the oligo spike-in and background probe sets (included in the control target content) across all of the arrays. According to Genisphere, oligo spike-in 2, 23, 29, 31 and 36 probe sets should present a value of more than 1000 intensity units to accept array quality. The miRNA arrays were detected using the Affymetrix detection algorithm, based on the non-parametric Wilcoxon rank-sum test, applied independently on each array and probe/probe set; a *p*-value greater than 0.06 stands for “not detected above background” [19]. For data normalization, the “default” method was used, obtaining log2 expression values (expression values data matrix) from the raw data (intensity values data matrix). Briefly, this method involved the following three steps: grouping the background probes intensities based on GCcontent, where the median intensity of each bin was the correction value for each probe with the same GC content; a quantile normalization and, finally, a median polish summarization. To obtain a single intensity value for each miRNA mapped on the log2 array, intensity measures for replicated spots were averaged.

#### 2.3.2. Agilent Human miRNA Microarray (V1)

Images were scanned using the Agilent Feature Extraction (AFE) software (version 11.0.1.1), obtaining the total gene signal (TGS) for all miRNAs on the array. Negative values were transformed by adding, for each array separately, the absolute value of the minimum TGS intensity on the array as extracted by the AFE + 2 before log2 transformation [20]. Data extracted from AFE were imported in the R environment [21] and processed using the AgimiRNA package, available in Bioconductor [22,23].

#### 2.3.3. Illumina HumanMI_V2

Raw data were processed using the proprietary BeadStudio software (Version 3.3.8). No background subtraction was performed. Probe-level data were summarized to obtain miRNA-level data, and then, a log2 transformation was applied.

#### 2.3.4. miRNA Selection and Normalization

The Affymetrix platform contained information on 7815 miRNAs, of which 847 (10.83%) were human, whereas Agilent platform content was of 961 miRNAs (851 human, 88.55%), and on the Illumina array 1145 miRNAs were detected, of which 858 (74.93%) were human miRNAs. Human miRNAs common to all platforms were selected according to their name and confirmed by a search on miRBase (Release 18, November 2011). Unlike other published works, miRNAs were not filtered on a detection basis, because such an approach could possibly introduce a bias in the results. In fact, some of the miRNAs that are filtered out because they are “switched-off” could show patterns of within- and/or between-platform disagreement in another experiment, where they are “turned-on”. This could possibly lead to an over-estimate of the level of reliability. Considering only human miRNAs should circumvent this issue and, at the same time, provide relevant information, since human miRNAs are commonly those that are of major interest in biomolecular investigation.

Moreover, no data normalization was performed. Almost all works that focused on comparing microarray platforms normalized their data (for instance, [15,16,24]), but this is a non-trivial issue that has to be carefully evaluated. As a matter of fact, to date, normalization for miRNA microarray has been largely debated, with results that have been somehow discordant [25,26,27,28,29], so that no “gold-standard” methods exists. Additionally, normalizing data in the context of assessing platform agreement poses other relevant problems. If data on two different platforms are normalized and then compared, then there is no way to discriminate between platform and normalization on the results of concordance/agreement/reproducibility assessment. A high level of between-platforms agreement, due not to the platforms themselves, but to the normalization used, might be found. On the other hand, the same normalization on different platforms could highlight patterns of discordance that cannot be ascribed to the platforms. Nonetheless, comparing un-normalized data exposes the risk of finding poor concordance, because of incidental batch effects occurring in the experiment, which may lead to an underestimate of the “true” agreement between platforms. In this paper, we have chosen to use non normalized data, so that we could assess the performance of different platforms “*per se*”. For the sake of comparison, data were also normalized with the quantile and loess algorithm, and results were compared to those obtained on non-normalized data.

### 2.4. Statistical Analysis

#### 2.4.1. Intra-Platform Reliability

To assess the reliability of the three miRNA microarray platforms, pair-wise concordance correlation coefficients [17] were computed for all possible pairs of technical replicates for all platforms, within each sample. The CCC $\rho_{c}$ between two series of *n* measurements *x* and *y* is defined as:

(1) $$\rho_{c}=\frac{2\sigma_{xy}}{\sigma_{x}^{2}+\sigma_{y}^{2}+{\left(\mu_{x}-\mu_{y}\right)}^{2}}=\frac{2\rho \sigma_{x}\sigma_{y}}{\sigma_{x}^{2}+\sigma_{y}^{2}+{\left(\mu_{x}-\mu_{y}\right)}^{2}}$$

where $\rho =\frac{\sigma_{xy}}{\sigma_{x}\sigma_{y}}=\frac{\sum_{i=1}^{n}\left(x_{i}-\bar{x}\right)\left(y_{i}-\bar{y}\right)}{\sqrt{\sum_{i=1}^{n}{\left(x_{i}-\bar{x}\right)}^{2}}\sqrt{\sum_{i=1}^{n}{\left(y_{i}-\bar{y}\right)}^{2}}}$ is the Pearson correlation coefficient between *x* and *y*, $\mu_{x}=\frac{\sum_{i=1}^{n}x_{i}}{n}$ and $\mu_{y}=\frac{\sum_{i=1}^{n}y_{i}}{n}$ are the sample means of *x* and *y* and $\sigma_{x}^{2}=\frac{\sum_{i=1}^{n}{\left(x_{i}-\bar{x}\right)}^{2}}{n-1}$ and $\sigma_{y}^{2}=\frac{\sum_{i=1}^{n}{\left(y_{i}-\bar{y}\right)}^{2}}{n-1}$ are the sample variances of *x* and *y*. Unlike the correlation coefficients, which only can give information about the existence of a linear relationship between two measurement methods, the CCC provides information on both precision (best-fit line) and accuracy (how far the best-fit line deviates from the concordance line) and is thus a better measure to assess platform reliability [30]. Additionally, the pairwise CCCs were combined within each sample and platform into an overall measure of reliability, the overall concordance correlation coefficient (OCCC) [31], a weighted mean of pairwise CCCs, which is defined as follows:

(2) $$\rho_{c}^{0}=\frac{\sum_{j=1}^{J-1}\sum_{k=j+1}^{J}\xi_{jk}\rho_{c}^{jk}}{\sum_{j=1}^{J-1}\sum_{k=j+1}^{J}\xi_{jk}}$$

where $\rho_{c}^{jk}$ is the standard Lin’s CCC between *j*-th and *k*-th replicate measurement series (in this study, these are the replicate arrays), and $\xi_{jk}$ are the weights, specific for each paired comparison:

(3) $$\xi_{jk}=\sigma_{j}^{2}+\sigma_{k}^{2}+{\left(\mu_{j}-\mu_{k}\right)}^{2}$$

Confidence intervals for the OCCC were computed using the bootstrap [32]. Specifically, 1000 bootstrap samples were extracted, and for each of these samples, sample means, variances, covariances, CCC and OCCC were computed. Then, using the empirical distribution of the bootstrap, estimates of the OCCC percentile confidence intervals at 95% were estimated.

To evaluate whether pairs of technical replicates are actually in agreement, the non-inferiority approach proposed by Liao and colleagues [33] for gene expression microarrays was followed. This approach consists of defining a threshold, or lower-bound, ${\rho_{c}}_{\left(CL\right)}$ reflecting the minimal value that the CCC should assume to conclude that two methods agree and then testing the following hypothesis:

(4) $$H_{0}:\rho_{c}\leq {\rho_{c}}_{\left(CL\right)}\;\;\;vs.\;\;\;H_{1}:\rho_{c}>{\rho_{c}}_{\left(CL\right)}$$

This can be done using the confidence intervals for both CCC and OCCC, interpreting the results as follows: if the lower confidence bound falls below ${\rho_{c}}_{\left(CL\right)}$, then the null hypothesis cannot be rejected and the two replicates cannot be said to be in agreement; otherwise, the two replicates are in agreement. To determine the value of $\rho_{CL}$, the authors define the minimum thresholds of precision and accuracy, and then, since the CCC can be seen as a product of a precision and accuracy term, ${\rho_{c}}_{\left(CL\right)}$ is computed as the product of these two thresholds. In their example, they propose a threshold of 0.90, yet in this paper, we have chosen to use the value of 0.96, according to the following formula:

(5) $${\rho_{c}}_{\left(CL\right)}=\frac{2\rho_{CL}}{\left(v_{CL}+v_{CL}^{-1}+u_{CL}^{2}\right)}=\frac{2*0.98}{0.9+0.9^{-1}+0.15^{2}}=0.9638\approx 0.96$$

where $v=\sigma_{1}/\sigma_{2}$ represents the scale shift between the two measurements series and $u=\left(\mu_{1}-\mu_{2}\right)/\sqrt{\sigma_{1}\sigma_{2}}$ is the location shift relative to the scale. The reason for the choice of these values is subjective, but in this case, there has been the attempt to be conservative: a higher value for $\rho_{CL}$ means a relationship between technical replicates as linear as possible, though leaving space for small departures due to ineffective probes or small experimental effects. On the other hand, increasing $v_{CL}$ to 0.9 is due to the fact that miRNA measurements are assumed to be less variable than gene expression, so that technical replicates may show very similar patterns of variability. Only $u_{CL}$ is unchanged, because the value proposed in [33] appeared reasonable also for miRNA microarrays.

#### 2.4.2. Between-Platform Agreement

In the microarray literature, concordance between platforms has been often studied using the correlation coefficient. Not only is this the wrong approach, but additionally, correlation coefficients are computed assuming that intensity/expression values do not suffer from any measurement error, thus leading to possible underestimates of the real level of correlation between platforms [34]. Here, agreement between platforms was evaluated using a modified version of the Bland–Altman approach. Such a modification, suggested by Liao *et al.* [35], allows one not only to assess whether two methods of measurement are concordant, but also to provide information on the eventual sources of disagreement. In a nutshell (greater details can be read in the original paper), the method involved the estimation, for each platform pair and separately for each sample, of a measurement error model, *i.e.*, a model where also the independent variable(s) *X* were assumed to be affected by uncertainty, of the form:

(6) $$Y_{i}=a_{0}+b_{0}X_{i}^{0}+\epsilon_{i}$$

(7) $$X_{i}=X_{i}^{0}+\delta_{i}$$

where $(X_{i}^{0},Y_{i}^{0}),i=1,...,n$ were the unobserved true values of the two measurement methods to be compared, *i.e.*, miRNAs intensities on the two platforms, and $\epsilon_{i}$ and $\delta_{i}$, were the i.i.d. error components of the model, which followed a normal distribution with the mean equal to 0 and variances equal to $\sigma_{\epsilon }^{2}$ and $\sigma_{\delta }^{2}$, respectively. To estimate this model, the ratio *λ* of the error variances of *Y* and *X* had to be known, possibly by means of replication or, when replication is not feasible, by setting it equal to 1, thus assuming equal error variances for both methods. In this study, both strategies were evaluated, using the technical replicates to estimate *λ* by fitting a linear model with the factor “replicate” as the covariate. The estimated residual variance was then used as the sample error variance for the platform. Once the parameters of the model were estimated, assuming that $Y-X∼N(a_{0}+\left(b_{0}-1\right)X^{0},\left(1+\lambda \right)\sigma_{\delta }^{2})$, modified versions of the agreement interval for Y−X proposed by Bland and Altman [36,37] were estimated according to the bias (fixed or proportional) needed to correct for when comparing two platforms, as follows:

**(a)**  **No bias:** ($a_{0}$ = 0, $b_{0}$ = 1)
  (8) $$\Delta =\left(-t_{1-\alpha /2,n-1}\sqrt{1+\lambda }{\hat{\sigma }}_{\delta },+t_{1-\alpha /2,n-1}\sqrt{1+\lambda }{\hat{\sigma }}_{\delta }\right)$$
**(b)**  **Fixed bias:** ($a_{0}$≠ 0, $b_{0}$ = 1)
  (9) $$\Delta =\left(a_{0}-t_{1-\alpha /2,n-1}\sqrt{1+\lambda }{\hat{\sigma }}_{\delta },a_{0}+t_{1-\alpha /2,n-1}\sqrt{1+\lambda }{\hat{\sigma }}_{\delta }\right)$$
**(c)**  **Proportional bias:** ($a_{0}$ = 0, $b_{0}\neq$ 1)
  (10) $$\Delta =\left(\left(b_{0}-1\right)X_{i}-t_{1-\alpha /2,n-1}\sqrt{1+\lambda }{\hat{\sigma }}_{\delta },\left(b_{0}-1\right)X_{i}+t_{1-\alpha /2,n-1}\sqrt{1+\lambda }{\hat{\sigma }}_{\delta }\right)$$
**(d)**  **Fixed and Proportional bias:** ( $a_{0}\neq$ 0, $b_{0}\neq$ 1)
  (11) $$\Delta =\left(a_{0}+\left(b_{0}-1\right)X_{i}-t_{1-\alpha /2,n-1}\sqrt{1+\lambda }{\hat{\sigma }}_{\delta },a_{0}+\left(b_{0}-1\right)X_{i}+t_{1-\alpha /2,n-1}\sqrt{1+\lambda }{\hat{\sigma }}_{\delta }\right)$$

where $X_{i}$ were the actual measured values of the method. Including only the parameter $a_{0}$ in the agreement interval meant that the two methods differ only by a “fixed” shift that did not depend on the value of $X_{i}$ (thus, fixed bias). Including only (or also) $b_{0}$, on the other hand, meant that the differences between the two methods increased proportionally with the increase of the value of measurement $X_{i}$ according to the value of the parameter $b_{0}$ itself (thus, proportional bias). Finally, let *n* be the number of subjects and 0<k<n; the methods were considered to be in agreement, after eventual bias correction, if no more than *k* subjects showed Y−X differences outside these intervals. The choice of *k* depends on the acceptable tolerance: the lower the tolerance for disagreeing subjects, the lower the value of *k*. The method thus involved estimation of the full model (both intercept and slope) and then evaluation of the most proper bias correction, according to inference on the parameter.

With respect to the common use, this method was used in a slightly different way. In a nutshell, commonly, there are *n* subjects on which specific biological quantities are measured using *k* measurement methods (k≥2), and the goal is to evaluate whether measurements from the *k* methods agree using information on *n* samples. Had this procedure been followed, each miRNA should have been evaluated separately (since the miRNA is the biological quantity of interest), and the intensities in the *n* samples (in our case, the two cell lines) should have been compared between the *k* platforms (in this case, 3), jointly, for both cell lines. Actually, the miRNAs were considered to be “subjects” and the entire profile of intensity on a platform to be the vector of measurements to be compared between different platforms (that is, k=3), separately, for each cell line.

All of the analyses were performed using R [21] and Bioconductor [23].

## 3. Results and Discussion

### 3.1. Data Description

**Figure 1:**
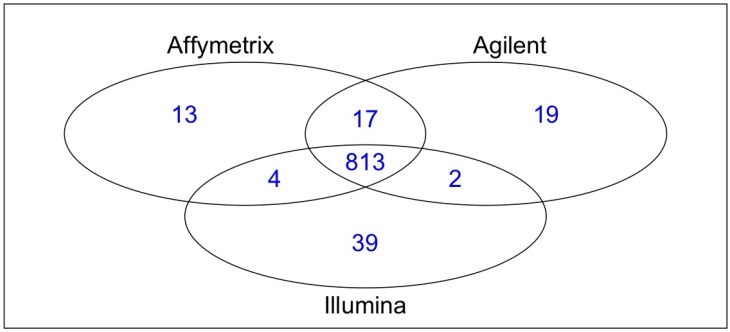
Venn diagram of common human miRNAs across platforms.

Analyses involved two samples (A498, a tumor line, and hREF, a pool of healthy tissues), each with three technical replicates for all platforms considered (Affymetrix GeneChip^©^ miRNA Array, Agilent Human miRNA Microarray (V1) and Illumina humanMI_V2). MiRNA selection described in the Experimental Section, resulted in a total of 813 human miRNAs considered for analysis, which account for 95.99% of human miRNAs on Affymetrix platforms, 95.53% on Agilent and 94.76% on Illumina (see Figure 1). Pairwise intersections of human miRNA lists revealed that the larger overlap occurred when Affymetrix and Agilent were considered (830 miRNAs, 97.99% of Affymetrix hsaand 97.53% of Agilent hsa), whereas Illumina showed a slightly poorer degree of overlap with both Affymetrix (817 miRNAs, 96.46% of Affymetrix and 95.22% of Illumina) and Agilent (815 miRNAs, 95.77% of Agilent and 94.99% of Illumina).

**Figure 2:**
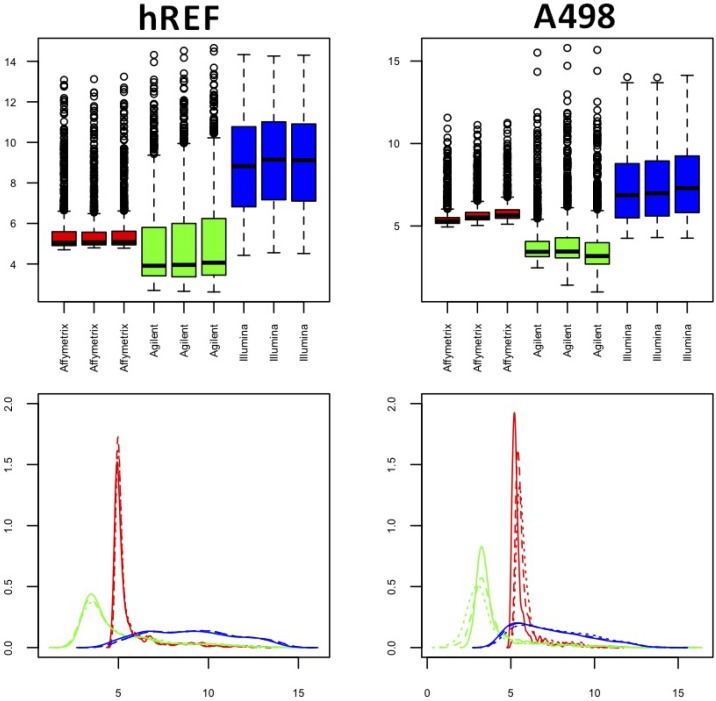
Box and density plots for both samples. The left column refers to hREF and the right column to A498. Plots refer to non-normalized log2-transformed data. (**Lower panels**) Solid lines represent the technical replicate labeled as 1 in the datasets, whereas dashed lines and dotted lines represent technical Replicate 2 and 3, respectively.

Density plots and boxplots for both samples in Figure 2 showed that the distribution of intensity values of common miRNAs was very different between platforms, with a marked skewness for Affymetrix and Agilent (red and green) and peaks of density for relatively low values. The Illumina platform was characterized by a slightly more symmetrical behavior. Notably, the first replicate of sample A498 had some technical problems for both Affymetrix and Agilent (solid red and green lines, lower right panel), whereas no similar pattern was seen for Illumina. In fact, the quantile distribution appeared to be quite different between technical replicates for these two platforms, despite similar inter-quartile ranges (see Table 1), suggesting that systematic bias had occurred in the profiling of the sample. The same plots were produced for normalized data and showed quite a different distribution, at least in terms of location, in particular for Illumina platform, whose right tail was relevantly reduced (see Supplementary Material, Figures S1 and S2).

**Table 1:** Quartiles and inter-quartile range for the three platforms.

		*Affymetrix*				*Agilent*				*Illumina*			
*Sample*	*Array*	*25th*	*50th*	*75th*	*IQR*	*25th*	*50th*	*75th*	*IQR*	*25th*	*50th*	*75th*	*IQR*
	1	4.907	5.044	5.592	0.685	3.415	3.911	5.804	2.389	6.821	8.830	10.772	3.951
*hREF*	2	4.943	5.066	5.570	0.627	3.366	3.955	5.998	2.632	7.168	9.150	11.015	3.847
	3	4.943	5.087	5.615	0.672	3.449	4.065	6.242	2.793	7.103	9.117	10.907	3.804
	1	5.160	5.285	5.508	0.348	3.139	3.432	4.065	0.926	5.492	6.863	8.782	3.290
*A498*	2	5.375	5.524	5.827	0.452	3.066	3.448	4.287	1.221	5.606	6.973	8.942	3.336
	3	5.468	5.651	5.983	0.515	2.690	3.175	3.988	1.298	5.812	7.294	9.246	3.433

### 3.2. Intra-Platform Reliability

Results for the pairwise CCCs and the overall CCC for each sample and platform using common human miRNAs are reported in Table 2. Globally, the hREF sample showed better features of reliability, in particular for the Agilent and Illumina platform (OCCC: 0.994 (CI 95%: 0.993–0.995) and 0.994 (CI 95%: 0.994–0.995), respectively). On the other hand, the A498 sample showed lower values of OCCC, which dramatically decreased when considering the Affymetrix platform (0.927, CI 95%: 0.906–0.941). According to the non-inferiority threshold of 0.96 defined in the Experimental Section, Agilent and Illumina could be consistently declared repeatable across the two samples considered, whereas Affymetrix resulted in repeatable only for sample hREF.

As a means of comparison to previous studies, these results were obtained also on data filtered using the detection call approach, *i.e.*, by removing all of the miRNAs that were called “present” on no more than two samples (see Table 3). This information was available only for Agilent and Illumina and led to a reduced dataset consisting of 347 human miRNAs. It can be noticed that estimates were mostly unchanged for the Illumina platform, though with a slight improvement after data filtering, whereas for Agilent, the pattern was not clear, though the differences appeared to be larger for A498, where filtering led to some relevant improvements, both in the pair-wise CCC and in the OCCC.

**Table 2:** CCC and Overall CCC with bootstrap 95%CI (common miRNAs) CCCs and the OCCC were computed on the 813 miRNAs common across all platforms considered for the study, and bootstrap 95%CI for the Overall Concordance Correlation Coefficient were computed using 1000 bootstrap samples and the percentile method [32].

		*Affymetrix*			*Agilent*			*Illumina*		
*Sample*	*Pair*	*CCC*	*OCCC*	*CI 95%*	*CCC*	*OCCC*	*CI 95%*	*CCC*	*OCCC*	*CI 95%*
	1-2	0.988			0.997			0.991		
*hREF*	1-3	0.993	0.992	(0.990–0.993)	0.989	0.994	(0.993–0.995)	0.993	0.994	(0.994–0.995)
	2-3	0.994			0.995			0.999		
	1-2	0.935			0.975			0.996		
*A498*	1-3	0.888	0.927	(0.906–0.941)	0.970	0.975	(0.969–0.979)	0.981	0.989	(0.987–0.991)
	2-3	0.961			0.978			0.989		

**Table 3:** Concordance correlation coefficient (CCC) and overall CCC with bootstrap 95% CI (detection call filtering). CCCs and the OCCC were computed on the 347 miRNAs common across Illumina and Agilent platforms after detection call filtering (present on at least three samples), and bootstrap 95% CI for the overall CCC were computed using 1000 bootstrap samples and the percentile method [32].

		*Agilent*			*Illumina*		
*Sample*	*Pair*	*CCC*	*OCCC*	*CI 95%*	*CCC*	*OCCC*	*CI 95%*
	1-2	0.996			0.988		
*hREF*	1-3	0.978	0.989	(0.987–0.990)	0.994	0.993	(0.992–0.994)
	2-3	0.992			0.998		
	1-2	0.974			0.997		
*A498*	1-3	0.988	0.983	(0.979–0.986)	0.987	0.992	(0.990–0.994)
	2-3	0.986			0.992		

CCCs and OCCC using all human miRNAs and all miRNAs (human and non-human) were also evaluated on each platform (see Supplementary Material, Tables S1 and S2). When considering all of the human miRNAs on each platform (847 for Affymetrix, 851 for Agilent and 858 for Illumina; Table S1), results were almost identical to the situation with only common miRNAs used, the only difference lying in the Affymetrix platform for sample A498, which showed an OCCC of 0.93 (CI 95%: 0.911–0.943), a slightly higher value than those seen for common miRNAs. Using all of the miRNAs measured on the platform (7815 for Affymetrix, 921 for Agilent and 1145 for Illumina) returned values that confirmed patterns of agreement already depicted in Table 2 and Table S1, with the exception of line A498 for Affymetrix, which showed an increase, both in point and interval estimates for the OCCC (0.954, CI 95%: 0.950–0.957, see Table S2). The Agilent platform on line A498, on the contrary, showed similar patterns of moderate-to-poor agreement throughout all miRNA selection strategies.

Additionally, the normalization effect was evaluated by estimating CCC and overall CCC on data normalized according to quantile and loess normalization [38], which resulted in a general increase of both pairwise and overall concordance for both samples and for all platforms. In particular, also sample A498 profiled with Affymetrix showed good patterns of repeatability, with its CI 95% lying above the threshold of 0.96. Detailed results were reported in Tables S3 and S4 of the Supplementary Material.

### 3.3. Between-Platform Agreement

To perform agreement evaluation, miRNA intensities were averaged across technical replicates for each array and both samples. Then, pairwise array agreements were evaluated in terms of miRNA lying within the modified agreement interval described in the Experimental Section. Estimates of the measurement error model for error-variance ratio *λ* equal to one, presented in Table 4, show that the relationship between Agilent and Illumina was the one that is closest to the agreement line with intercept zero and slope one for both samples. On the other hand, models that include the Affymetrix platform for line A498 showed a very negatively large intercept (−12.4128 and −17.4064), which possibly reflected the technical bias already highlighted in the previous section. However, if line hREF is considered, Affymetrix was confirmed to be the array deviating most from the line of perfect agreement with both Illumina and Agilent, whereas these two showed patterns very close to concordance (slope = 1.0925, CI 95%: 1.0371–1.1479). Since the confidence intervals for the intercept and the slope suggested an intercept different from zero and a slope different from one for all comparisons in both samples, the agreement intervals following Formula 11 were built.

**Table 4:** Estimates of the linear measurement error model, *λ* = 1.

		$a_{0}$		$b_{0}$	
*Sample*	*Pair*	*Estimate*	CI 95%	*Estimate*	CI 95%
	Agilent *vs*. Affymetrix	−6.1037	(−6.4471, −5.7603)	1.9610	(1.8964, 2.0255)
*hREF*	Illumina *vs*. Affymetrix	−4.7630	(−5.5006, −4.0254)	2.4418	(2.3472, 2.5363)
	Illumina *vs*. Agilent	3.6033	(3.3791, 3.8274)	1.0925	(1.0371, 1.1479)
	Agilent *vs*. Affymetrix	−14.2358	(−16.3792, −12.0923)	3.1409	(2.9815, 3.3004)
*A498*	Illumina *vs*. Affymetrix	−17.4064	(−18.6406, −16.1722)	4.2889	(4.1679, 4.4098)
	Illumina *vs*. Agilent	2.1916	(1.7773, 2.6058)	1.3254	(1.2407, 1.4100)

**Figure 3:**
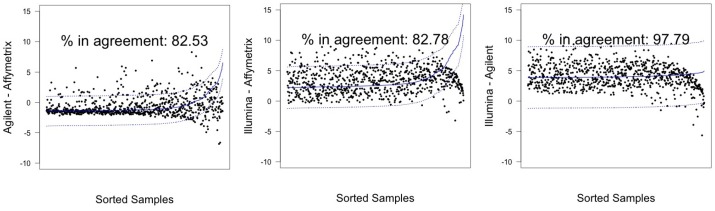
Agreement intervals for line hREF, λ=1. (**A**) The comparison Agilent-Affymetrix; (**B**) the comparison Illumina-Affymetrix; and (**C**) Illumina-Agilent. The samples/miRNAS have been plotted in ascending order according to their value on the second platform in the y-axis label, so that the x-axis only contains a progressive value from one to 813 according to such ordering.

**Figure 4:**
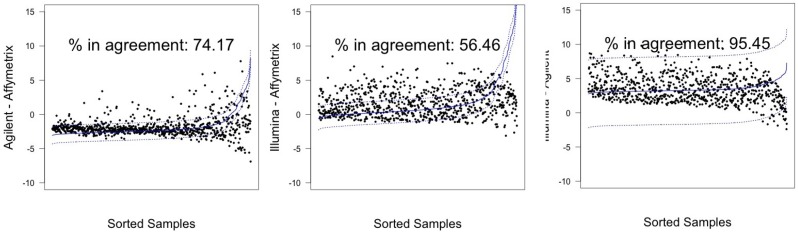
Agreement intervals for line A498, λ=1. (**A**) The comparison Agilent-Affymetrix; (**B**) the comparison Illumina-Affymetrix; and (**C**) Illumina-Agilent. The samples/miRNAs have been plotted in ascending order according to their value on the second platform in the y-axis label, so that the x-axis only contains a progressive value from one to 813 according to such ordering.

In Figure 3 and Figure 4, graphical results for lines A498 and hREF, respectively, were reported. When a value *k* of tolerance equal to 0.05×813 ≃ 41 was set, thus considering the platforms to be in agreement if no more than *k* measurements lay outside these intervals, the pair Illumina-Agilent was the only one that could be said to be in agreement for both samples. In fact, 97.79% (CI 95%: 96.52–98.68) of miRNAs for hREF and 95.45% (CI 95%: 93.78–96.78) of miRNAs for A498 were concordant between the two platforms (795 and 776 miRNAs in agreement for cell lines hREF and A498, respectively). On the other hand, the remaining pairs showed poor patterns of concordance. The Affymetrix platform on line A498 was poorly concordant with both Agilent and Affymetrix, possibly because of the issues previously described, but also for line hREF, it showed a poor degree of agreement. In particular, only 82.53% (CI 95%: 79.75–85.08) and 82.78% (CI 95%: 80.01–85.31) of miRNAs, corresponding to 671 and 673, were found to lie within the agreement interval for comparison to Agilent and Illumina, respectively.

These results relied on the assumption that the ratio of the error variances from the two methods being compared was equal to one. The main advantage of this assumption was that previous knowledge of the error variances was not required; however, it was likely to be violated when the methods to be compared had very different analytical properties, such as microarray platforms (see, for instance, Figure 2). The technical replicates were used to fit a random effects model for each combination of cell line and array, estimated the error variance as the residual error of the model itself and computed the parameter *λ* as the ratio of the error variance of *Y* and *X* (see Table 5). The confidence intervals for *λ* did not include one in any comparison, thus suggesting a different measurement error for each platform.

**Table 5:** Estimates of *λ* and CI 95%. Values were obtained as the ratio of $\sigma_{\epsilon }^{2}$ (error variance of Y) and $\sigma_{\delta }^{2}$ (error variance of X), estimated via random effects models.

*Sample*	*Pair*	*λ*	CI 95%
	*Agilent-Affymetrix*	2.608	2.409–2.824
hREF	*Illumina-Affymetrix*	2.935	2.711–3.178
	*Illumina Agilent*	1.125	1.039–1.218
	*Agilent-Affymetrix*	4.125	3.810–4.466
A498	*Illumina-Affymetrix*	5.576	5.150–6.037
	*Illumina-Agilent*	1.352	1.248–1.463

Model parameters estimates when *λ* is estimated according to a random effects model are reported in Table 6. The estimates of the slopes and of the intercepts suggested that also in this case, the interval to be preferred should be the one described in Formula 11.

**Table 6:** Estimates of the linear measurement error model, *λ* estimated.

		$a_{0}$		$b_{0}$	
*Sample*	*Pair*	*Estimate*	CI 95%	*Estimate*	CI 95%
	Agilent *vs*. Affymetrix	−7.3495	(−8.1460, −6.5529)	2.1806	(2.0824, 2.2789)
*hREF*	Illumina *vs*. Affymetrix	−7.0221	(−9.3909, −4.6533)	2.8402	(2.6707, 3.0096)
	Illumina *vs*. Agilent	3.4477	(3.2018, 3.6936)	1.1235	(1.0655, 1.1816)
	Agilent *vs*. Affymetrix	−12.4128	(−12.9575, −11.8681)	2.8265	(2.7461, 2.9068)
*A498*	Illumina *vs*. Affymetrix	−20.3938	(−29.7127, −11.0749)	4.8042	(4.4718, 5.1366)
	Illumina *vs*. Agilent	1.5756	(0.9998, 2.1514)	1.4804	(1.3806, 1.5802)

**Figure 5:**
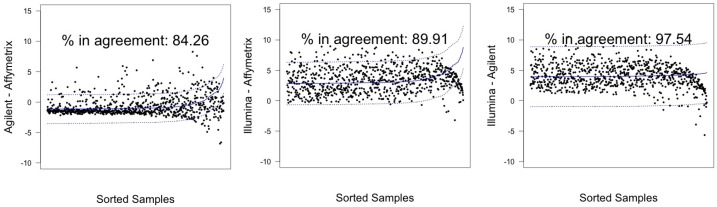
Agreement intervals for line hREF, *λ* estimated. (**A**) The comparison Agilent-Affymetrix; (**B**) the comparison Illumina-Affymetrix; and (**C**) Illumina-Agilent. The samples/miRNAs have been plotted in ascending order according to their value on the second platform in the y-axis label, so that the x-axis only contains a progressive value from one to 813 according to such ordering.

**Figure 6:**
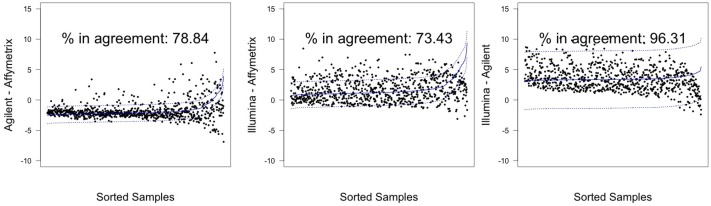
Agreement intervals for line A498, *λ* estimated. (**A**) The comparison Agilent-Affymetrix; (**B**) the comparison Illumina-Affymetrix; and (**C**) Illumina-Agilent. The samples/miRNAs have been plotted in ascending order according to their value on the second platform in the y-axis label, so that the x-axis only contains a progressive value from one to 813 according to such ordering.

Illumina and Agilent showed the best patterns of concordance for both samples, resulting in a percentage of miRNAs in agreement exceeding the 95% threshold previously discussed: 97.54% (CI 95%: 96.23–98.49) for hREF and 96.3% (CI 95%: 94.77–97.50) for A498, corresponding to 793 and 783 miRNA (see Figure 5 and Figure 6).

In general, estimating the value of *λ* led to an increased number of miRNAs within the determined agreement interval, in particular for the Affymetrix-related comparisons (see Table 7).

Performing the same analysis on detection call filtered data (thus, only on 347 miRNAs and comparing Agilent and Illumina platforms only) led to similar results, though with some improvement for line A498. In particular, if *λ* was set to one, the proportion of concordant miRNAs was equal to 97.69% (CI 95%: 95.51–99.00) for line hREF and to 98.56% (CI 95%: 96.67–99.53) for line A498. Estimating the error variance ratio (0.629, CI 95%: 0.557–0.710 for hREF; 0943, CI 95%: 0.835–1.065 for A498) led to slightly improved results, the proportion of concordant miRNAs being equal to 99.14% for hREF (CI 95%: 96.67–99.53) and to 98.56% (CI 95%: 97.49–99.82) for A498.

The same analysis was performed also on quantile and loess normalized data, with contrasting results. For sample A498, there was a general increase in the performance of all platforms, yet only Agilent and Illumina could be considered to be concordant (net of bias correction), whereas for sample hREF, there was a general decrease, at least when *λ* = 1 was considered, in the proportion of concordant miRNAs. This difference was substantially relevant when Affymetrix and Illumina were compared (see Tables S8 and S12 in the Supplementary Material). The fact that differences between normalized and un-normalized data were more relevant when *λ* was assumed to be one could be due to the effect of the normalization procedure on the error variance ratio: in particular, when normalization is performed, there is a use of information carried by the data that can lead to a reduction in the residual error variance and in the ratio *λ* and, eventually, to more stable results and, thus, more concordant measurements. On the other hand, differences between platforms are already taken into account when *λ* is estimated, so that a normalization procedure could only limitedly improve results.

**Table 7:** miRNA in agreement between arrays. Number (*n*) and proportion (%) of miRNAs lying in the different agreement intervals, estimated according to the measurement error model parameters estimated by setting *λ* = 1 and by estimating it via random effects models. Confidence intervals for the proportions were computed using the Clopper–Pearson exact method [39].

		λ=1		*λ* Estimated	
*Sample*	*Comparison*	%(CI95%)	*n*	%(CI95%)	*n*
	*Agilent-Affymetrix*	82.53 (79.75–85.08)	671	84.26 (81.57–86.70)	685
*hREF*	*Illumina–Affymetrix*	82.78 (80.01–85.31)	673	89.91 (87.64–91.90)	731
	*Illumina-Agilent*	97.79 (96.52–98.68) ^†^	795	97.54(96.23–98.49) ^†^	793
	*Agilent-Affymetrix*	74.17 (71.02–77.15)	603	78.84 (75.87–81.60)	641
*A498*	*Illumina-Affymetrix*	56.46 (52.97–59.90)	459	73.43 (70.25–76.44)	597
	*Illumina-Agilent*	95.45 (93.78–96.78)	776	96.31 (94.77–97.50)	783

^†^: the platform pair is in agreement.

Results for model parameter and *λ* estimation on normalized data are available in the Supplementary Material, from Tables S5 to S7 for quantile normalization and from S9 to S11 for loess normalization.

## 4. Conclusions

Studies evaluating high-throughput platform reliability by means of correlation coefficients, both Pearson and Spearman [15], are often seen in the literature. Many of these studies claim that high correlation coefficients imply high reliability. This misuse of the correlation coefficients has already been outlined in the microarray literature [30]; however, several studies still use these measures to assess the repeatability or reproducibility of high-throughput platforms.

In this study, both the issue of within-platform reliability and between-platform agreement of three miRNA microarray platforms were discussed. Results have highlighted that Agilent and Illumina were the platforms showing the best pattern of both reliability and agreement, whereas Affymetrix appeared to have some technical problem. In terms of reliability, the OCCC for line A498 was relevantly lower than that for line hREF for all platforms and for all sets of miRNAs considered (common human miRNAs, human miRNAs array-wise, all miRNAs). Overall, Illumina (OCCC from 0.989 to 0.994) showed better performances with respect to Agilent (from 0.975 to 0.994) and, in particular, Affymetrix (from 0.927 to 0.993), and this was better seen for line A498. Since this line suffered from technical issues on Affymetrix and, partially, also on Agilent, the related OCCC possibly underestimated the “true” degree of reproducibility of the platforms, which could be better appreciated with line hREF, where it ranged from 0.992 (CI 95%: 0.990–0.993) to 0.993 (CI 95%: 0.993–0.994), with values almost identical to those of Agilent and Illumina, thus suggesting also in this case a good performance of the array. These results, however, only referred to the “repeatability” of the assay, meaning that technical replicates were performed under “conditions where independent test results are obtained with the same method on identical test items in the same laboratory by the same operator using the same equipment within short intervals of time”. Conversely, reproducibility implies “conditions where test results are obtained with the same method on identical test items in different laboratories by different operators using different equipment”, as defined by the ISO [40]. This specification is relevant, since studies claiming to have evaluated the reproducibility of arrays actually evaluated only their repeatability [15], whereas reproducibility assessment necessarily requires the involvement of multiple laboratories, as in the MAQCProject [41].

Assessing agreement between different methods of measurement is a task that has rarely been addressed in the microarray literature, where the focus has always been more on the evaluation of a linear relationship between platforms or with “gold-standard” assays, such as qPCR, mainly via computation of correlation coefficients. Such an approach has often been biased by the selection of miRNAs used to do the computations, in that often, only miRNAs concordantly detectable between platforms [15,16] were chosen, possibly leading to an overestimate of the real level of correlation between arrays. To avoid this issue, human miRNAs common (*i.e.*, matched by name) on all of the platforms considered for the experiment were used.

By setting a “strong” threshold at 95%, equivalent to 772 miRNAs, it was found that Agilent and Illumina arrays were concordant according to the agreement interval derived from the estimation of the measurement error model, irrespective of the method for choosing the value of *λ*. The choice of this threshold was subjective and should depend on the issue at hand; the present choice was based on the fact that it is likely that a few miRNAs exist that do not agree because of unwanted technical issues not attributable to the platform itself, but also to the need to reduce the number of false positives (*i.e.*, falsely concordant platforms). This last point is crucial in the field of microarrays, since the comparability of results from different platforms as a tool for validating a laboratory’s own results has gained much relevance in omic research. As a matter of fact, the basic assumption is that the two platforms are “linked” in that what they convey about the profile of intensity/expression for a sample is similar, the net of the different measurement and the analytic scale of the platform itself.

There is, however, a possible confounding effect in these results that was not possible to control, *i.e.*, the site where the arrays were processed. As described in the Experimental Section, Affymetrix arrays were processed at a laboratory (say LAB1), Agilent and Illumina at another one (say LAB2), so that when the comparison between Affymetrix and the other platforms is done, different laboratories and different platforms are compared in a completely confounded way. In this perspective, it is not clear to what extent the laboratory effect encompasses the platform effect, *i.e.*, how much the differences between Affymetrix and the other platforms were due to different sites of processing and how much to actual assay differences. Similarly, it could not be exclude that the high degree of concordance between Illumina and Agilent could be sharpened by this confounding effect. One alternative could have been to have all of the arrays processed at a single site, so that the shared laboratory effect would cancel out, yet this was not possible, for the facilities involved in the experiment did not have all of the instrumentation needed for performing the experiment with all of the arrays. Thus, though being confident in the validity of our results, we cannot discriminate between “true” agreement, *i.e.*, concordance due to real similarity between platforms, and “technical” agreement, *i.e.*, concordance or discordance related to the lab where the platform was processed.

**Figure 7:**
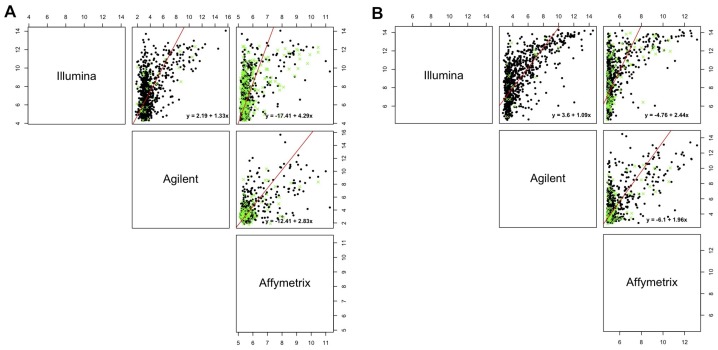
Measurement error model fit. The red line represents fitted values of the measurement error model and green crosses represent miRNAs not in agreement after bias correction. For simplicity, we represent the model fit for λ=1 separately for line A498 (**A**) and hREF (**B**).

Notably, the measurement error model considered here was just one of the possible models that could be fitted. Scatterplots of Figure 7 show the fitted lines for each of the comparisons when λ=1. It is easy to note that the linearity of the relationship between discordant platforms can be questioned, so that the identified differences could be due to a non-linearity in it or to a lack-of-fit of the regression line, which can be accounted for by considering different functional forms for the *x* variable, both in a linear and in a non-linear context.

To conclude, these results show that Agilent and Illumina were the most concordant platforms showing good patterns of agreement, whereas Affymetrix-related comparisons showed poor agreement for both lines. For line A498, this could be explained by technical issues on one replicate, whereas for line hREF, this could possibly be due to a non-linear relationship between the arrays, whose biological or technical source we were not able to ascertain. This suggests considering different functional forms to achieve a better characterization of the relationship.

The power of the proposed method is that it can be used to assess agreement in various contexts and, though supposing a simple linear relation exists between arrays, allows one to estimate it in terms of model parameters corrected for the presence of measurement error, an issue that is often neglected in microarray studies.

## Supplementary Files

Supplementary File 1
