# Supplementary material for: Assessing Agreement between miRNA Microarray Platforms
Source: Microarrays (Basel). 2014 Dec 12;3(4):302–21. doi: 10.3390/microarrays3040302 (PMC4979053; doi:10.3390/microarrays3040302)

Supplementary Information

Supplementary Tables

Table S1. CCC and overall CCC with bootstrap 95% CI (all human miRNAs).

		Affymetrix			*Agilent*			*Illumina*		
Sample	*Pair*	*CCC*	*OCCC*	*CI 95%*	*CCC*	*OCCC*	*CI 95%*	*CCC*	*OCCC*	*CI 95%*
	1–2	0.988			0.997			0.991		
hREF	1–3	0.993	0.992	(0.990–0.993)	0.989	0.994	(0.993–0.995)	0.993	0.994	(0.994–0.995)
	2–3	0.994			0.995			0.999		
	1–2	0.939			0.975			0.996		
A498	1–3	0.893	0.93	(0.911–0.943)	0.970	0.975	(0.969–0.979)	0.981	0.989	(0.987–0.991)
	2–3	0.961			0.962			0.989		

Table S2. CCC and overall CCC with bootstrap 95% CI (all miRNAs).

		Affymetrix			*Agilent*			*Illumina*		
Sample	*Pair*	*CCC*	*OCCC*	*CI 95%*	*CCC*	*OCCC*	*CI 95%*	*CCC*	*OCCC*	*CI 95%*
	1–2	0.990			0.997			0.990		
hREF	1–3	0.995	0.993	(0.993–0.994)	0.990	0.994	(0.994–0.995)	0.992	0.994	(0.993–0.994)
	2–3	0.996			0.996			0.999		
	1–2	0.959			0.977			0.996		
A498	1–3	0.928	0.954	(0.950–0.957)	0.970	0.976	(0.970–0.980)	0.981	0.988	(0.987–0.990)
	2–3	0.976			0.980			0.988		

Table S3. CCC and overall CCC with bootstrap 95% CI (quantile normalization).

		Affymetrix			*Agilent*			*Illumina*		
Sample	*Pair*	*CCC*	*OCCC*	*CI 95%*	*CCC*	*OCCC*	*CI 95%*	*CCC*	*OCCC*	*CI 95%*
	1–2	0.992			0.998			0.999		
hREF	1–3	0.994	0.994	(0.992–0.995)	0.998	0.998	(0.997–0.998)	0.998	0.999	(0.998–0.999)
	2–3	0.995			0.998			0.999		
	1–2	0.972			0.991			0.998		
A498	1–3	0.967	0.971	(0.963–0.977)	0.993	0.993	(0.991–0.994)	0.995	0.996	(0.995–0.997)
	2–3	0.973			0.994			0.995		

Table S4. CCC and overall CCC with bootstrap 95% CI (loess normalization).

		Affymetrix			*Agilent*			*Illumina*		
Sample	*Pair*	*CCC*	*OCCC*	*CI 95%*	*CCC*	*OCCC*	*CI 95%*	*CCC*	*OCCC*	*CI 95%*
	1–2	0.989			0.998			0.998		
hREF	1–3	0.991	0.992	(0.989–0.993)	0.997	0.998	(0.997–0.998)	0.998	0.998	(0.998–0.999)
	2–3	0.994			0.998			0.999		
	1–2	0.983			0.990			0.998		
A498	1–3	0.978	0.981	(0.976–0.985)	0.992	0.992	(0.990–0.994)	0.995	0.996	(0.995–0.997)
	2–3	0.981			0.994			0.995		

Table S5. Estimates of the linear measurement error model, $\lambda = 1$ (quantile normalization).

		a_0		b_0	
Sample	*Pair*	*Estimate*	**CI 95%**	*Estimate*	**CI 95%**
	Agilent *vs.* Affymetrix	−8.1304	(−8.4960, −7.7648)	2.2040	(2.1378, 2.2702)
hREF	Illumina *vs.* Affymetrix	−8.3585	(−9.0694, −7.6476)	2.8994	(2.8071, 2.9918)
	Illumina *vs.* Agilent	2.9633	(2.7599, 3.1668)	1.1761	(1.1204, 1.2319)
	Agilent *vs.* Affymetrix	−9.0705	(−9.5760, −8.5650)	2.3613	(2.2836, 2.4391)
A498	Illumina *vs.* Affymetrix	−11.2364	(−12.5018, −9.9710)	3.4002	(3.2772, 3.5233)
	Illumina *vs.* Agilent	2.8587	(2.3850, 3.3325)	1.2101	(1.1250, 1.2952)

Table S6. Estimates of λ and CI 95% (quantile normalization). Values obtained as a ratio of σ_ϵ^2 (error variance of Y) and σ_δ^2 (error variance of X), estimated via random effects models.

Sample	*Pair*	λ	**CI 95%**
	Agilent-Affymetrix	3.116	2.878–3.374
hREF	*Illumina-Affymetrix*	3.891	3.594–4.213
	Illumina Agilent	1.249	1.153–1.352
	Agilent-Affymetrix	3.132	2.892–3.390
A498	*Illumina-Affymetrix*	3.827	3.535–4.144
	Illumina-Agilent	1.222	1.129–1.323

Table S7. Estimates of the linear measurement error model, λ estimated (quantile normalization).

		a_0		b_0	
Sample	***Pair***	***Estimate***	**CI 95%**	***Estimate***	**CI 95%**
	Agilent *vs*. Affymetrix	−5.6388	(−5.7125, −5.5650)	1.7689	(1.7392, 1.7987)
hREF	Illumina *vs*. Affymetrix	−3.0838	(−3.1592, −3.0085)	1.9784	(1.9483, 2.0084)
	Illumina *vs*. Agilent	3.2250	(3.0627, 3.3873)	1.1179	(1.0680, 1.1677)
	Agilent *vs*. Affymetrix	−5.8006	(−5.8875, −5.7138)	1.7923	(1.7600, 1.8245)
A498	Illumina *vs*. Affymetrix	−3.1551	(−3.2488, −3.0614)	1.9938	(1.9604, 2.0273)
	Illumina *vs*. Agilent	3.3203	(2.9651, 3.6755)	1.1075	(1.0338, 1.1812)

Table S8. Number (n) and proportion ($\%$) of miRNA lying within the agreement intervals, estimated according to the measurement error model parameters estimated by setting λ = 1 and by estimating it via random effects models. Data were normalized according to the quantile normalization algorithm. Confidence intervals for the proportions were computed using the Clopper–Pearson exact method.

		$\lambda = 1$		λ **Estimated**	
Sample	***Comparison***	$\%$ $(CI95\%)$	***n***	$\%$ $(CI95\%)$	***n***
	Agilent-Affymetrix	79.46 (76.52, 82.19)	646	81.55 (78.71, 84.16)	663
hREF	*Illumina-Affymetrix*	71.34 (68.1, 74.43)	580	80.93 (78.06, 83.58)	658
	Illumina-Agilent	96.43 (94.92, 97.6)	784	96.19 (94.63, 97.39)	782
	Agilent-Affymetrix	78.84 (75.87, 81.6)	641	82.41 (79.62, 84.97)	670
A498	*Illumina-Affymetrix*	70.48 (67.21, 73.6)	573	83.89 (81.18, 86.35)	682
	Illumina-Agilent	98.52 (97.44, 99.24) †	801	98.77 (97.75, 99.41) †	803

†: the platform pair is in agreement.

Table S9. Estimates of the linear measurement error model, λ = 1 (loess normalization).

		a_0		b_0	
Sample	***Pair***	***Estimate***	**CI 95%**	***Estimate***	**CI 95%**
	Agilent *vs*. Affymetrix	−8.5056	(−8.8715, −8.1397)	2.2852	(2.2189, 2.3515)
hREF	Illumina *vs*. Affymetrix	−8.7511	(−9.4867, −8.0155)	2.9867	(2.8927, 3.0807)
	Illumina *vs*. Agilent	3.0661	(2.8601, 3.2720)	1.1540	(1.0984, 1.2096)
	Agilent *vs*. Affymetrix	−8.6769	(−9.1779, −8.1759)	2.2718	(2.1943, 2.3493)
A498	Illumina *vs*. Affymetrix	−11.0793	(−12.3307, −9.8279)	3.3492	(3.2267, 3.4718)
	Illumina *vs*. Agilent	2.6593	(2.1942, 3.1245)	1.2559	(1.1700, 1.3418)

Table S10. Estimates of λ and CI 95% (loess normalization). Values obtained as ratio of σ_ϵ^2 (error variance of Y) and σ_δ^2 (error variance of X), estimated via random effects models.

Sample	*Pair*	λ	**CI 95%**
	Agilent-Affymetrix	3.306	3.054–3.579
hREF	*Illumina-Affymetrix*	4.021	3.714–4.354
	Illumina Agilent	1.216	1.123–1.317
	Agilent-Affymetrix	2.967	2.740–3.212
A498	*Illumina-Affymetrix*	3.779	3.490–4.091
	Illumina-Agilent	1.274	1.176–1.379

Table S11. Estimates of the linear measurement error model, λ estimated (loess normalization).

		a_0		b_0	
Sample	*Pair*	***Estimate***	**CI 95%**	***Estimate***	**CI 95%**
	Agilent *vs.* Affymetrix	−5.8600	(−5.9285, −5.7916)	1.8232	(1.7945, 1.8518)
hREF	Illumina *vs.* Affymetrix	−3.1706	(−3.2438, −3.0973)	2.0121	(1.9825, 2.0418)
	Illumina *vs.* Agilent	3.2998	(3.1301, 3.4694)	1.1030	(1.0525, 1.1534)
	Agilent *vs.* Affymetrix	−5.6135	(−5.7061, −5.5208)	1.7369	(1.7036, 1.7703)
A498	Illumina *vs.* Affymetrix	−3.1723	(−3.2666, −3.0780)	1.9687	(1.9350, 2.0023)
	Illumina *vs.* Agilent	3.2046	(2.8815, 3.5276)	1.1301	(1.0585, 1.2017)

Table S12. Number (n) and proportion (%) lying within the agreement intervals, estimated according to the measurement error model parameters by setting λ = 1 and via random effects models. Data were normalized according to the loess normalization algorithm. Confidence intervals for the proportions were computed using the Clopper–Pearson exact method.

		$\lambda = 1$		λ **Estimated**	
Sample	*Comparison*	$\%\ (CI95\%)$	***n***	$\%\ (CI95\%)$	***n***
	Agilent-Affymetrix	79.21 (76.26, 81.95)	644	81.18 (78.32, 83.81)	660
hREF	*Illumina-Affymetrix*	71.83 (68.60, 74.90)	584	82.90 (80.14, 85.43)	674
	Illumina-Agilent	96.06 (94.49, 97.29)	781	95.82 (94.2, 97.09)	779
	Agilent-Affymetrix	79.95 (77.03, 82.65)	650	83.27 (80.53, 85.77)	677
A498	*Illumina-Affymetrix*	68.76 (65.45,71.93)	559	83.03 (80.27, 85.54)	675
	Illumina-Agilent	98.4 (97.28, 99.15) †	800	98.52 (97.44, 99.24) †	801

† the platform pair is in agreement.

Supplementary Figures

Figure S1. Box and density plots for both samples. The left column refers to hREF and the right column to A498. Plots refer to quantile normalized log2-transformed data. (**Lower panels**) The solid line represents the technical replicate labeled as 1 in the datasets, whereas the dashed line and dotted line represent technical Replicates 2 and 3, respectively.

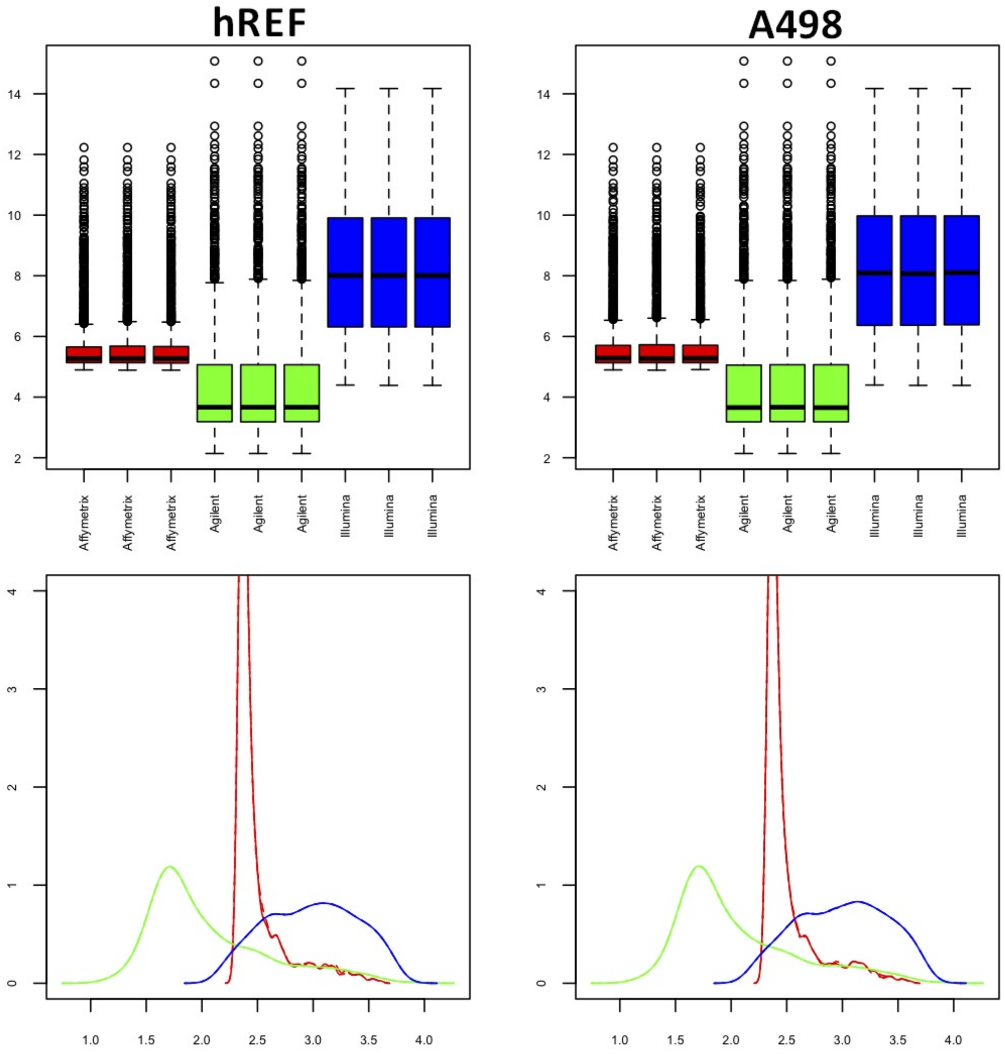

Figure S2. Box and density plots for both samples. The left column refers to hREF and the right column to A498. Plots refer to loess normalized log2-transformed data. (**Lower panels**) The solid line represents the technical replicate labeled as 1 in the datasets, whereas the dashed line and dotted line represent technical Replicates 2 and 3, respectively.

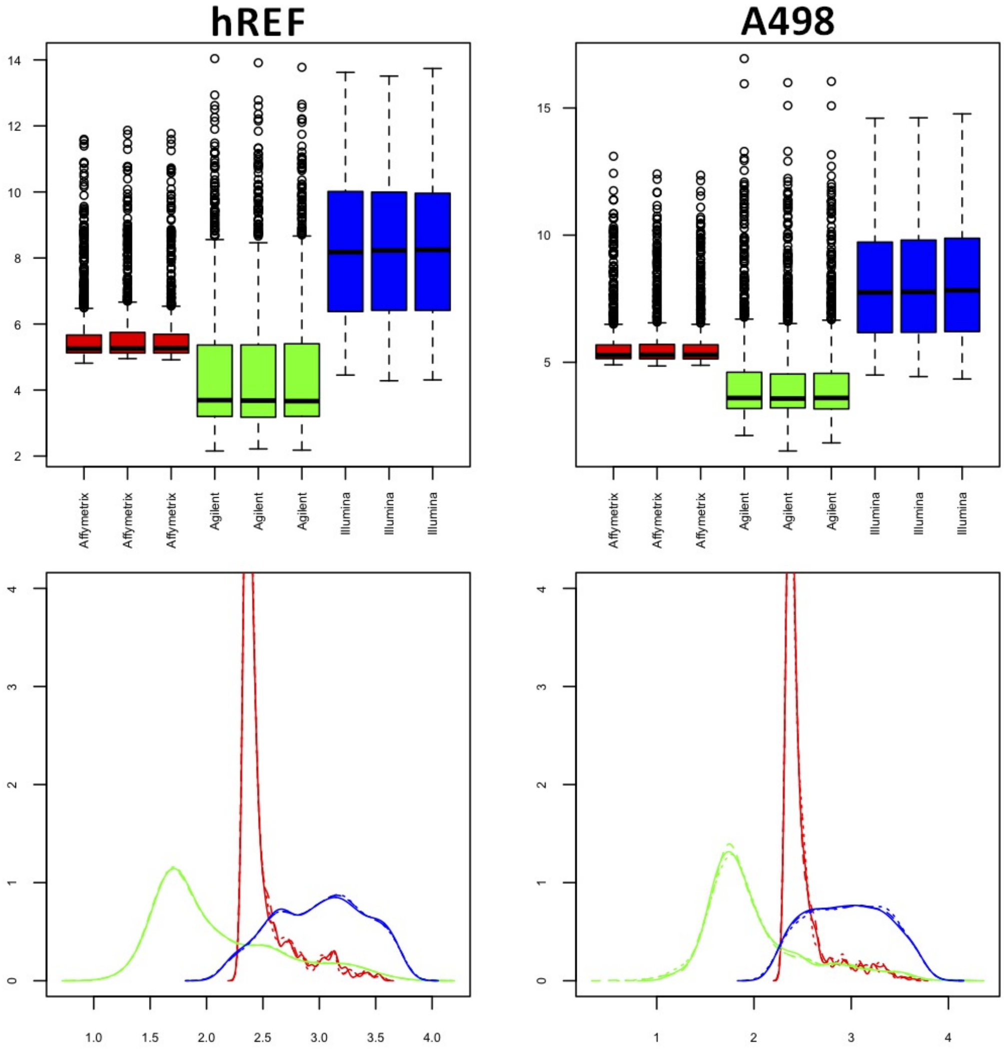

Supplement: Supplementary File 1 [file microarrays-03-00302-s001.pdf]
